# *Plasmodium falciparum* infection dysregulates placental autophagy

**DOI:** 10.1371/journal.pone.0226117

**Published:** 2019-12-05

**Authors:** Flávia Afonso Lima, André Barateiro, Jamille Gregório Dombrowski, Rodrigo Medeiros de Souza, Douglas de Sousa Costa, Oscar Murillo, Sabrina Epiphanio, Lígia Antunes Gonçalves, Claudio Romero Farias Marinho

**Affiliations:** 1 Department of Parasitology, Institute of Biomedical Sciences, University of São Paulo, São Paulo, Brazil; 2 Multidisciplinary Centre, Federal University of Acre, Acre, Brazil; 3 Department of Clinical and Toxicological Analysis, School of Pharmaceutical Sciences, University of São Paulo, São Paulo, Brazil; Instituto Rene Rachou, BRAZIL

## Abstract

*Plasmodium (P*.*) falciparum* malaria during pregnancy has been frequently associated with severe consequences such as maternal anemia, abortion, premature birth, and reduced birth weight. Placental damage promotes disruption of the local homeostasis; though, the mechanisms underlying these events are still to be elucidated. Autophagy is a fundamental homeostatic mechanism in the natural course of pregnancy by which cells self-recycle in order to survive in stressful environments. Placentas from non-infected and *P*. *falciparum*-infected women during pregnancy were selected from a previous prospective cohort study conducted in the Brazilian Amazon (Acre, Brazil). Newborns from infected women experienced reduced birth weight (*P* = 0.0098) and placental immunopathology markers such as monocyte infiltrate (*P* < 0.0001) and IL-10 production (*P* = 0.0122). The placentas were evaluated for autophagy-related molecules. As a result, we observed reduced mRNA levels of *ULK1* (*P* = 0.0255), *BECN1* (*P* = 0.0019), and *MAP1LC3B* (*P* = 0.0086) genes in placentas from *P*. *falciparum*-infected, which was more striking in those diagnosed with placental malaria. Despite the protein levels of these genes followed the same pattern, the observed reduction was not statistically significant in placentas from *P*. *falciparum*-infected women. Nevertheless, our data suggest that chronic placental immunopathology due to *P*. *falciparum* infection leads to autophagy dysregulation, which might impair local homeostasis during malaria in pregnancy that may result in poor pregnancy outcomes.

## Introduction

Malaria is still considered an important global health problem despite the continuous worldwide effort to eliminate malaria. During 2017, a concerning number of about 219 million cases and approximately half a million deaths have been reported as a result of *Plasmodium* spp. infection [1]. Among them are pregnant women, which constitute a risk group for developing malaria in pregnancy (MiP). *Plasmodium (P*.*) falciparum* is responsible for the most severe clinical manifestations of malaria during pregnancy, namely maternal anemia, abortion, preterm delivery, fetal growth restriction, and reduced birth weight [2,3].

The poor pregnancy outcomes are most likely to occur when placental malaria (PM) is settled by the sequestration of *P*. *falciparum-*infected erythrocytes within the placenta. Placental malaria is defined by the accumulation of parasite or *Plasmodium* by-products, such as hemozoin, in the intervillous space [4]. The sequestration occurs through the preferential binding of the *P*. *falciparum* erythrocyte membrane protein 1 (PfEMP1) variant VAR2CSA to the chondroitin sulfate A (CSA), abundantly expressed by the syncytiotrophoblast [5–7]. In response to the parasite accumulation, chemokines are produced and recruit monocytes to the site, which orchestrate a local inflammatory response with massive cytokine production [8–12]. Meanwhile, considerable histopathologic alterations occur in the placenta, such as the formation of syncytial nuclear aggregates and fibrinoid necrosis as a reflection of the extensive inflammatory process [13,14]. These immunologic and histologic events compromise placental homeostasis. The imbalance of physiologic mechanisms involved in placental angiogenesis, hormonal production, and nutrient transport to the growing fetus, result in the frequently observed impaired fetal development [15]. However, the molecular mechanisms by which *falciparum* malaria impairs placental homeostasis are still to be fully determined.

One of the key processes in maintaining cellular and tissue homeostasis is autophagy, which galvanizes metabolic and immunologic adaptation in response to a highly diverse plethora of stress-inducing agents. Succinctly, intracellular isolation of a double-membrane complex occurs and enwraps specific and selected cargo for degradation (autophagosome formation), which later will fuse to lysosomes (autophagolysosome). This will ultimately lead to the digestion of previously selected cargo and promotes nutrients recycling and organelle turnover [16]. Exogenous or endogenous signs like high nutritional and energetic demands, hypoxia, organelle-associated stress, cell growth, lineage differentiation, inflammation, survival/death programs, and infection can activate or inhibit autophagy in a tightly regulated manner [16,17]. Autophagy has been shown to be involved in embryonic and placental development. Also, alterations in placental autophagic profile have been observed during preeclampsia, fetal growth restriction, and inflammation-induced preterm delivery that implicated the success of pregnancy [18].

Noteworthy, in *P*. *falciparum*-infected women, placental monocyte infiltrate levels at delivery have been shown to be predictive of placental autophagy dysregulation in areas of high and stable malaria transmission [19]. On the contrary, the Brazilian Amazon basin is an area of unstable transmission that contributes with approximately 42% of malaria cases in the Americas, where *falciparum* infections during pregnancy are promptly treated [20,21]. Despite the rapid intervention and clearance of systemic infection, placental stress and lesions perpetuate until parturition, which probably dictate the poor gestational outcomes [14,22]. Hence, placental mechanisms of homeostasis like autophagy might be dysregulated due to chronic inflammation and damage caused by *P*. *falciparum* infection. Herein, we show that relevant autophagy-associated gene transcripts are downregulated, while protein levels present a downward trend in placentas from *P*. *falciparum-*infected women, which are likely to be associated with chronic placental inflammation and tissue damage.

## Materials and methods

### Ethical approval and participants consent

Ethical clearance was provided by the committee for research of the University of São Paulo (Plataforma Brasil, CAAE: 86696718.5.0000.5467 and 59989316.1.0000.5467), according to Resolution n° 466/12 of Brazilian National Health Committee. All the study participants enrolled in a main prospective cohort study between January 2013 and April 2015 or their legal guardians (if minors) gave written informed consent, authorizing the usage of the collected data and biological samples for further research purposes. Data and samples were acquired from a biorepository at the Institute of Biomedical Sciences, University of São Paulo–ICB/USP, which is under the responsibility of Professor Claudio R. F. Marinho. Authors declared to maintain the confidentiality of the collected data to be used for research purposes only. The main study was conducted in accordance with the Declaration of Helsinki and is registered in the Brazilian Clinical Trials Registry as RBR-3yrqfq.

### Studied population

Non-infected (45) and *P*. *falciparum*-infected (43) pregnant women were selected according to maternal age, gravidity, and gestational age from a prospective study involving 600 women from a malaria-endemic region located in the Brazilian Amazon. Participants were recruited at the *Hospital da Mulher e da Criança do Juruá* (*HMCJ*, *Cruzeiro do Sul*, *Acre*, *Brasil*) between January 2013 and October 2014, during their first antenatal care visit. The women on both groups were selected based on their clinical records and habits, excluding women with other comorbidities such as hypertension, pre-eclampsia, diabetes, or diagnosed infectious diseases (i. e. syphilis, hepatitis or HIV), as well as illicit drugs, smoking, or alcohol consumption. More detailed information about the study design and settings are described elsewhere [22].

### Data and sample collection

Obstetric data was collected from all participants at the time of parturition. Additionally, peripheral blood was collected, and smears were performed to diagnose *P*. *falciparum* infection during pregnancy. Confirmed infections were promptly treated according to the Brazilian Ministry of Health guidelines [20]. Results were further confirmed by real-time PCR (PET-PCR, Photo-induced Electron Transfer-Polymerase Chain Reaction technique) [23]. At delivery, newborn’s anthropometric data and biological samples were collected, such as placental biopsies and blood. The blood was collected in heparin tubes and centrifuged to separate cells from plasma for further cytokine quantification. Placental tissue was preserved in RNA stabilizer (RNAlater, Life Technologies) or fixed in 10% buffered formalin and stored at– 80°C or 4°C, respectively, until being used for either molecular biology or histology procedures.

### Placental histology analysis

Fixed placental tissue was embedded in paraffin after being transported to the University of São Paulo. Tissue was then sliced in 5 μm sections and stained with Hematoxylin & Eosin (H&E) for the quantification of syncytial nuclear aggregates, fibrin deposition, and necrosis. The presence of parasite and hemozoin, the malarial pigment, were detected under polarized light microscopy and evaluated to identify the presence or absence of placental malaria, according to the classification described elsewhere [4]. Villous vascularity, leukocytes, and monocytes were assessed by quantifying CD31, CD45, and CD68 positive cells, respectively, using immunohistochemistry associated with the tissue microarray technique (TMA) developed at the *Hospital AC Camargo* (*São Paulo*, *Brazil*). Techniques mentioned above and quantification methods were performed as described elsewhere [14,22]. The analysis was conducted using the Zeiss Axio Imager M2 light microscope containing a Zeiss Axio Cam HRc (Zeiss, Oberkochen, Germany). Images were analyzed using ZEN 2 lite (Zeiss) and ImageJ software (http://imagej.nih.gov/ij).

### Placental cytokine quantification

Placental blood samples were processed before cytokine quantification. Briefly, plasma was separated from blood cells and cytokines IL-12, IL-8, TNF-α, IL-10, IL-6, and IL-1β were quantified using CBA Human Inflammatory Kit (BD Biosciences). Samples were analyzed using a FACSCalibur cytometer and the FCAP Array software V3.0.1 (BD Biosciences). The procedure was conducted according to the manufacturer’s instructions.

### Quantification of autophagy-related genes using quantitative PCR

Total RNA was isolated and purified from 100 mg of placental tissue using TRIzol and Purelink RNA Mini Kit (ThermoFisher). One microgram of RNA was converted into cDNA using the High-Capacity cDNA Reverse Transcription Kit (ThermoFisher). The expression of the genes *ULK1* (Hs00177504), *BECN1* (Hs01011598), and *MAP1LC3B* (Hs00797944) was quantified using the TaqMan Gene Expression Master Mix (ThermoFisher) and the QuantStudio 12K Flex Real-Time PCR System (ThermoFisher). The gene *GAPDH* (Hs99999905) was used as an endogenous control in a multiplex PCR, simultaneously with the target genes. All experiments were performed according to the manufacturer’s instructions.

### Quantification of autophagy-related proteins using western blot

Placental tissue preserved in RNAlater was mechanically macerated in RIPA lysis buffer (ThermoFisher) previously supplemented with Pierce^TM^ Protease inhibitor (ThermoFisher). Samples were centrifuged and supernatants were collected, from which proteins were quantified using Pierce^TM^ BCA Protein Assay (ThermoFisher). After quantification, proteins were separated by electrophoresis in a 10% (ULK1 and BECLIN1) or 15% (LC3) polyacrylamide gel and transferred to a PVDF membrane containing 0.45 μm (ULK1 and BECLIN1) or 0.2 μm (LC3) pores. Membranes were blocked with a 5% milk solution diluted in TBS-T (TBS 1x/Tween20 0.05%) for 2 hours. After blocking, membranes were incubated overnight at 4°C with a rabbit IgG antibody against ULK1 (1:3000, EPR4885, ab128859, Abcam), BECLIN1 (1:10000, EPR19662, ab207612, Abcam) and LC3 (1:5000, L7543, Sigma-Aldrich) diluted in 1% milk solution in TBS-T. In the next day, membranes were washed and incubated with goat anti-rabbit IgG HRP antibody (AP307P, Merck-Millipore) diluted 1:30000 (ULK1) or 1:20000 (BECLIN1) in 1% milk solution in TBS-T for 1 hour at room temperature. For LC3, the secondary goat anti-rabbit IgG HRP antibody (ab6721, Abcam) was used at a concentration of 1:10000. β-ACTIN was used as an endogenous control for further normalization of the bands related to the proteins of interest. Briefly, the same PVDF membranes used for target proteins were blocked with a 5% milk solution diluted in TBS-T for 2 hours at room temperature. Incubation with mouse IgG antibody against β-ACTIN (1:40000, AC-15, NB600-501, Novus Biologicals) diluted in 1% milk solution in TBS-T was performed after blocking for another 2 hours at room temperature. Secondary antibody recognition was done using a goat anti-mouse HRP IgG (AP308P, Merck-Millipore) diluted 1:20000 in 1% milk solution in TBS-T. Incubation was performed for 1 hour at room temperature. After, membranes were revealed by chemiluminescence using the Clarity Western ECL Substrate (Bio-Rad). Images were acquired at the ChemiDoc XRS+ (Bio-Rad) and Image Lab software V4.0 (Bio-Rad). Bands were further quantified using the ImageJ software (http://imagej.nih.gov/ij) by measuring band pixel density without being treated or overexposed. Values were then normalized to pixel density from β-ACTIN control bands. The β-ACTIN and the target protein blotting were performed in the same membrane. Placental protein samples from 14 non-infected and 14 *P*. *falciparum*-infected women were selected and used in two separated western blots gels/membranes to achieve a more reliable sample size. Non-infected and *P*. *falciparum* groups were uniformly separated across gels. Electrophoresis was performed simultaneously, blotting in parallel, and images were acquired at the same time/exposure using the ChemiDoc XRS+ (additional information and original gels/membranes can be found in supporting information S1 Fig).

### Statistical analysis

Groups were evaluated using the D’Agostino-Pearson test to infer about the normality distribution of the data. Further, differences between groups were analyzed using Student’s t-test (parametric data set) or Mann-Whitney U test (non-parametric data set) for single comparisons, and Kruskal-Wallis test with Dunn’s post-test for multiple comparisons. For correlation analysis, Spearman’s rank-order non-parametric correlation test was applied to determine the association between studied variables. Two-sided *P* values were used at a significance level of 0.05. Statistical data analysis was performed using Stata V14.2 (StataCorp) and GraphPad Prism V6.01 (USA) software.

## Results

### General characteristics of the participants

A group of non-infected (NI—45) and *P*. *falciparum*-infected (Pf-INF—43) pregnant women were selected from a major prospective study conducted at the *Hospital da Mulher e da Criança do Juruá* (Cruzeiro do Sul, Acre, Brazil). The women on both groups were selected based on their clinical records and habits, excluding women with other comorbidities or infectious diseases, as well as users of illicit drugs, smoking, or alcohol consumption. The women for both groups were selected based on identical maternal and gestational age, and gravidity. Therefore, these parameters did not present differences between groups **(Table 1)**. Predictably, *P*. *falciparum* infection during pregnancy was shown to lead to a significant reduction of maternal body mass index (BMI) (median [IQR], 21.51 [20.45–25.51] kg/m^2^ vs 20.58 [19.47–22.26] kg/m^2^, *P* = 0.0128), placental weight (median [IQR], 592.60 [519.20–661.80] g vs 545.20 [488.20–587.10], *P* = 0.0211), and newborns birth weight (median [IQR], 3.25 [3.03–3.69] kg vs 3.12 [2.93–3.35] kg, *P* = 0.0098) when compared to their non-infected counterpart **(Table 1** and S1 Table**)**. However, we did not observe alterations in the newborn/placental weight ratio, often considered as the best indicator of impaired gestational development [24].

**Table 1:** Characteristics of pregnant women enrolling the study according to infection status.

	NON-INFECTED		*P*. *falciparum*-INFECTED			*P*. *falciparum*-INFECTED PM-			*P*. *falciparum*-INFECTED PM+		
Characteristics, median (IQR)		n		n	*P* value		n	*P* value*		n	*P* value*
Obstetric											
**Maternal age, years**	24.00 (18.00–28.50)	45	23.00 (17.00–28.00)	43	0.7812 ^#^	23.00 (20.00–28.00)	15	>0.9999	21.50 (17.00–27.75)	28	>0.9999
**Gravidity**	1.00 (1.00–3.00)	45	2.00 (1.00–3.00)	39	0.9484 ^†^	2.00 (1.00–2.75)	12	>0.9999	1.00 (1.00–3.25)	26	>0.9999
**Gestational age, weeks**	39.00 (39.00–40.00)	45	39.00 (38.00–40.00)	43	0.2728^#^	39.00 (38.00–40.00)	15	>0.9999	39.00 (38.00–40.00)	28	0.8504
**Body mass index, kg/m**^**2**^	21.51 (20.45–25.51)	44	20.58 (19.47–22.26)	40	***0*.*0128*** ^†^	20.97 (19.46–22.29)	15	0.4289	20.32 (19.39–22.33)	25	***0*.*0484***
**Placental weight, g**	592.60 (519.20–661.80)	45	545.20 (488.20–587.10)	39	***0*.*0211***^#^	527.70 (488.20–569.30)	15	0.1022	564.80 (477.3–614.90)	25	0.4315
**Newborn weight, kg**	3.25 (3.03–3.69)	45	3.12 (2.93–3.35)	43	***0*.*0098***^#^	3.12 (2.93–3.32)	15	0.3006	3.16 (2.92–3.40)	28	0.2412
**Newborn/placental weight ratio**	5.71 (5.05–6.01)	44	5.76 (5.32–6.17)	40	0.3449 ^#^	5.70 (5.62–6.12)	15	0.7363	5.66 (5.13–6.22)	26	>0.9999
**Placental histology**											
**Hemozoin, %**											
**No**	100	45	39.53	17	ND	100	15	ND	7.14	2	ND
**Mild**	0	0	32.56	14	ND	0	0	ND	50.00	14	ND
**Moderate**	0	0	27.91	12	ND	0	0	ND	42.86	12	ND
**Severe**	0	0	0	0	ND	0	0	ND	0	0	ND
**Syncytial nuclear aggregates**	12.00 (9.00–15.00)	44	14.00 (11.00–20.00)	43	***0*.*0266*** ^†^	12.00 (8.00–16.00)	15	>0.9999	16.50 (12.00–21.75)	28	***0*.*0192***
**Fibrin**	1.92 (1.83–2.42)	45	2.00 (1.92–2.83)	43	0.0777^#^	1.92 (1.92–2.75)	15	>0.9999	2.17 (1.92–2.90)	28	0.0547
**Necrosis**	6.77 (4.29–9.98)	45	7.96 (5.26–9.96)	43	0.8465 ^#^	8.55 (5.35–9.96)	15	>0.9999	7.13 (4.29–9.94)	28	>0.9999
**Vascularity (CD31+)**	4.00 (3.40–4.70)	43	4.40 (3.60–4.95)	38	0.1354 ^#^	4.35 (3.42–5.20)	12	>0.9999	4.40 (3.68–4.95)	26	0.3011
**Leukocytes (CD45+)**	13.00 (9.00–19.00)	42	20.00 (12.00–31.75)	42	***0*.*0012***^†^	19.00 (12.00–35.00)	15	***0*.*0330***	21.00 (11.00–31.00)	27	***0*.*0220***
**Monocytes (CD68+)**	4.00 (1.00–6.00)	42	7.00 (5.00–16.00)	41	***<0*.*0001*** ^†^	7.00 (5.00–16.00)	15	***0*.*0008***	7.00 (5.00–14.00)	27	***0*.*0002***
**Cytokines (pg/mL)**											
**IL-12**	3.92 (3.10–4.33)	45	3.60 (3.36–4.02)	39	0.4350^#^	3.72 (2.68–4.02)	15	>0.9999	3.59 (3.40–4.23)	24	>0.9999
**IL-8**	24.42 (14.50–44.22)	39	24.68 (15.55–52.29)	36	0.4682 ^†^	22.66 (14.09–35.36)	14	>0.9999	31.48 (16.95–79.63)	22	0.5477
**TNF-α**	5.46 (4.62–6.42)	44	5.27 (4.58–6.04)	35	0.4190 ^†^	4.95 (3.80–5.90)	14	0.5392	4.40 (4.71–6.17)	21	>0.9999
**IL-10**	3.66 (3.06–4.83)	42	4.61 (3.48–5.79)	36	***0*.*0122*** ^†^	4.69 (3.29–5.46)	15	0.3244	4.53 (3.66–6.40)	21	0.0583
**IL-6**	61.33 (21.94–102.30)	36	64.33 (27.55–157.60)	37	0.2926 ^†^	40.29 (16.99–101.30)	13	>0.9999	102.90 (40.73–169.20)	23	0.2307
**IL-1β**	4.88 (4.17–5.60)	42	4.73 (3.71–5.71)	36	0.4803 ^†^	4.14 (3.68–6.01)	15	0.7369	4.88 (3.96–5.74)	22	>0.9999

*P*. *falciparum*-INFECTED PM-, *Plasmodium falciparum*-infected pregnant women with placental malaria negative; *P*. *falciparum*-INFECTED PM+, *Plasmodium falciparum*-infected pregnant women with placental malaria positive; IQR, interquartile range; n, number of individuals. Bold values depict *P* values < 0.05.

^#^ Differences between NON-INFECTED and *P*. *falciparum*-INFECTED were evaluated using the Student’s t-test when data set fit normality after application of proper normality test.

^†^ Differences between NON-INFECTED and *P*. *falciparum*-INFECTED were evaluated using Mann-Whitney rank sum test when data sets did not fit normality, after application of proper normality test.

* Differences between NON-INFECTED and *P*. *falciparum*-INFECTED PM- or *P*. *falciparum*-INFECTED PM+ were evaluated using the non-parametric Kruskal-Wallis test with Dunn’s post-test.

Poor pregnancy outcomes are well known to be a consequence of considerable placental histological disorganization that leads to dysregulation of local homeostasis and consequently reduced newborns’ birth weight. Therefore, placental histologic evaluation revealed that placentas from Pf-INF women had a significant increase of syncytial nuclear aggregate (SNA) counts (median [IQR], 12.00 [9.00–15.00] vs. 14.00 [11.00–20.00], *P* = 0.0266), but no statistically significant differences of fibrin deposition, necrosis scores, nor placental vascularity **(Table 1)**.

Though, a massive infiltrate of leukocytes (CD45^+^ cells, median [IQR], 13.00 [9.00–19.00] vs 20.00 (12.00–31.75), *P* = 0.0012) and monocytes (CD68^+^ cells, median [IQR], 4.00 [1.00–6.00] vs 7.00 [5.00–16.00], *P* < 0.0001) were detected, which support the existence of an inflammatory environment in placentas from *P*. *falciparum*-infected women. However, the same conclusion cannot be taken from the placental cytokine analysis, which presented similar levels between NI and Pf-INF women. Yet, with an exception for IL-10 that abundantly accumulated in placentas from infected women (median [IQR], 3.66 [3.06–4.83] pg/mL vs 4.61 [3.48–5.79] pg/mL, *P* = 0.0122) **(Table 1)**. Interestingly, the Pf-INF data stratification by placental malaria status (as per recommendations elsewhere [4,22]) revealed that the reduction of maternal BMI and increase of SNA were promoted by the occurrence of placental malaria (Pf-INF PM+) **(Table 1)**. All these deleterious outcomes were present at delivery, despite the frequent absence of peripheral and placental parasitemia observed at term (S1 Table).

These results support the hypothesis of a chronic pathology in Pf-INF placentas associated with changes of the tissue morphology and extensive inflammation, which might be linked to local homeostasis imbalance.

### Autophagy-related genes are downregulated in placentas from *P*. *falciparum-*infected women

**Fig 1:**
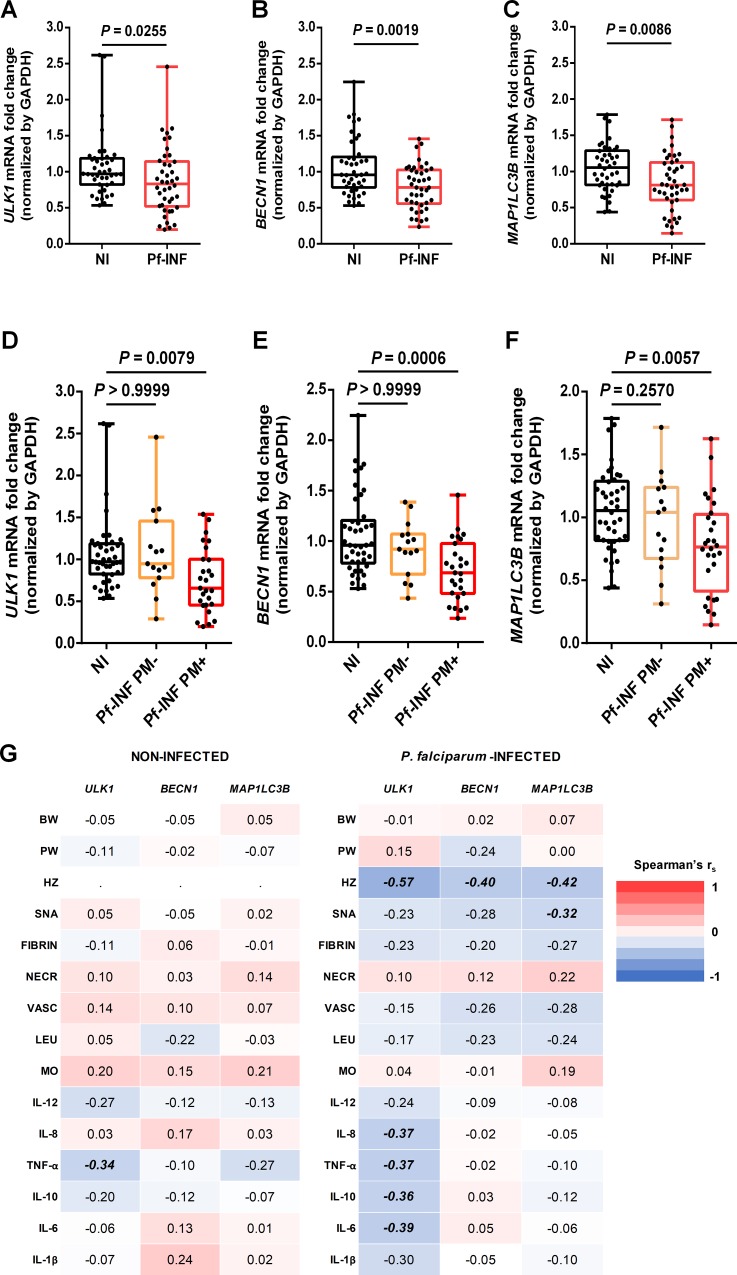
Effect of *P*. *falciparum* infection during pregnancy in placental autophagy-related genes expression. **(A-C)** Placental mRNA expression of the selected autophagy-related genes *ULK1* **(A)**, *BECN1* **(B),** and *MAP1LC3B* **(C)** in non-infected (NI) and *P*. *falciparum*-infected (Pf-INF) women. Results represent qPCR estimates relative to NI and normalized by *GAPDH*, endogenous control. Data sets on placental mRNA levels from *P*. *falciparum*-infected women were afterwards stratified according to placental malaria (PM) status as placental malaria negative (Pf-INF PM-) or positive (Pf-INF PM+) and plotted for *ULK1* **(D)**, *BECN1* **(E),** and *MAP1LC3B* **(F)**. **(G)** Heatmap matrixes of NI and Pf-INF placentas pairwise Spearman correlation coefficients (r_s_) between mRNA levels and histologic and immunologic parameters. Data are represented as whiskers boxplots where the bottom and the top of the box are the first and third quartiles, the line inside the box is the median and the whiskers represent the minimum and the maximum values **(A-F)**, and heatmaps containing r_s_ in a colour range from dark blue (r_s_ = -1) to dark red (r_s_ = 1) with statistically significant correlations enhanced as bold values **(G)**. The differences between groups were determined by the Mann-Whitney U test **(A-C)**, Kruskal-Wallis test with Dunn’s post-test for multiple comparisons **(D-F)**, and Spearman’s rank-order non-parametric test for correlations **(G)**. *P* values < 0.05 were considered as representing statistically significant differences. BW–birth weight, PW–placental weight, HZ–hemozoin, SNA–syncytial nuclear aggregates, NECR–necrosis, VASC–vascularity, LEU–leukocytes, MO–monocytes.

The increased number of monocytes/leukocytes and other observed indicators of tissue damage (i.e., syncytial nuclear aggregates and fibrin), together with the significant reduction of placental and newborns weight allowed to speculate that classical mechanisms of homeostasis maintenance might be dysregulated. Since autophagy is a key process in cellular and tissue homeostasis, we sought to investigate whether placental autophagy was altered in Pf-INF women. Therefore, the expression of principal autophagy-related genes was evaluated in placentas from both NI and Pf-INF women. *ULK1*, *BECN1*, and *MAP1LC3B* **(Fig 1A–1C)** were selected due to their critical involvement in the autophagic process. ULK1 and BECLIN1 (*BECN1*) are core-forming proteins of major autophagy complexes responsible for its initiation, while LC3 (*MAP1LC3B*) is pivotal in ensuring the process elongation by leading to autophagosome formation and maturation [16,18].

Quantification of mRNA levels by qPCR revealed a statistically significant downregulation of *ULK1* (*P* = 0.0255), *BECN1* (*P* = 0.0019) and *MAP1LC3B* (*P* = 0.0086) in placentas from Pf-INF women **(Fig 1A–1C)**. Stratification of Pf-INF women according to placental malaria status revealed that statistically significant differences were mostly explained by Pf-INF with placental malaria (Pf-INF PM+) in which mRNA levels of *ULK1* (*P* = 0.0079), *BECN1* (*P* = 0.006), and *MAP1LC3B* (*P* = 0.0057) were significantly downregulated when compared to NI placentas **(Fig 1D–1F)**. Interestingly, transcript levels from placentas of Pf-INF PM- presented no statistically significant differences from NI.

Additionally, Spearman’s rank-order correlation coefficient (r_s_) analysis revealed associations between placental histologic and immunologic parameters with mRNA of autophagy-related genes **(Fig 1G)**. Thus, gene expression variation in placentas from Pf-INF women showed moderate negative correlations with part of placental parameters measured across this study. The *ULK1* mRNA levels in Pf-INF women negatively correlated with the cytokines IL-8 (r_s_ = -0.37, *P* = 0.0276), IL-10 (r_s_ = -0.36, *P* = 0.0356), and IL-6 (r_s_ = -0.39, *P* = 0.0199), which is indicative of a moderate association between lower *ULK1* expression and augmented cytokine levels. Interestingly, the malaria pigment, hemozoin (HZ),—showed a moderate negative correlation with the mRNA levels of the three genes: *ULK1* (r_s_ = -0.57, *P* < 0.0001), *BECN1* (r_s_ = -0.40, *P* = 0.0088), and *MAP1LC3B* (r_s_ = -0.42, *P* = 0.0053). Besides, a moderate inverse correlation was also found between *MAP1LC3B* expression and syncytial nuclear aggregates (SNA) counts (r_s_ = -0.32, *P* = 0.0378) (full detailed analysis at S2 Table). Altogether, these results suggest that parasite by-products, inflammatory mediators, and tissue damage are associated with a transcriptional dysregulation of autophagy-related genes, which is likely to be more prominent when chronic placental malaria is patent.

### Autophagy-related protein levels were diminished in placentas from *P*. *falciparum*-infected women

**Fig 2:**
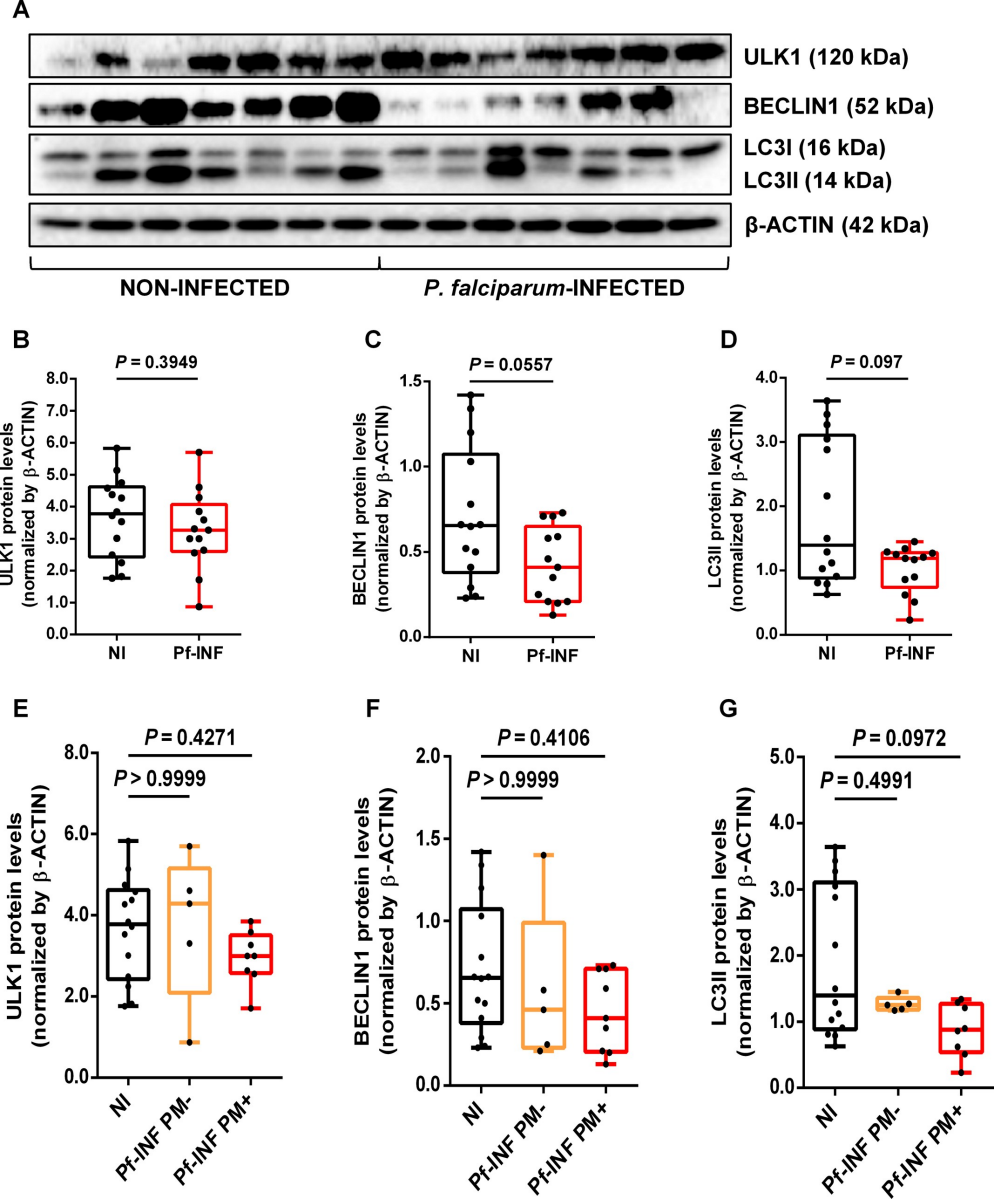
Levels of the autophagy-related proteins are diminished in *P*. *falciparum*-infected placentas. Placental autophagy-related protein levels measured in non-infected (NI) and *P*. *falciparum*-infected (Pf-INF) women. **(A)** Representative image of cropped western blotting of ULK1 (120 kDa), BECLIN1 (52 kDa), LC3I, and LC3II (16 and 14 kDa, respectively), and corresponding β-ACTIN (42 kDa) (full-length blots at S1 Fig). Western blot was performed at two to three independent experiments. **(B-D)** Semi-quantification by densitometry analysis of the ULK1 **(B)**, BECLIN1 **(C)**, and LC3II **(D)** protein levels. Semi-quantification of the target proteins was performed in different blots without image manipulation and normalized to the corresponding β-ACTIN (endogenous control), which blotting was conducted in the same membrane. Data sets on placental autophagy protein levels from *P*. *falciparum*-infected women were afterwards stratified according to placental malaria (PM) status as placental malaria negative (Pf-INF PM-) or positive (Pf-INF PM+) and plotted for ULK1 **(E)**, BECLIN1 **(F)**, and LC3II **(G).** Data are represented as whiskers boxplots where the bottom and the top of the box are the first and third quartiles, the line inside the box is the median, and the whiskers represent the minimum and the maximum values **(B-G)**. Statistical analysis was performed using the Mann-Whitney U test **(B-D)** or Kruskal-Wallis test with Dunn’s post-test for multiple comparisons **(E-G)**. *P* values < 0.05 were considered as representing statistically significant differences.

Despite clear transcriptional modulation of placental autophagy-related genes by *P*. *falciparum* infection, the question of whether these modifications were extended to corresponding proteins still prevailed. Therefore, approximately 14 placenta samples from the NI and Pf-INF groups were randomly selected for western blot analysis **(Fig 2A)** (full-length blots at S1 Fig). We have analyzed the proteins ULK1 (120 kDa), BECLIN1 (52 kDa), and both the soluble LC3I (16 kDa) and lipidated autophagosome-associated LC3II (14 kDa) isoforms. An apparent reduction of the BECLIN1 and LC3II protein levels from Pf-INF placentas **(Fig 2A)** was observed in the western blot; though, quantification by densitometry revealed no statistically significant differences (ULK1—*P* = 0.3949; BECLIN1—*P* = 0.0557; LC3II—*P* = 0.097) **(Fig 2B–2D)**.

Similarly to the autophagy-related gene expression, the results suggest that the reduction of these three principal autophagy protein mediators observed in the infected group was conferred by the Pf-INF PM+ placentas **(Fig 2E–2G)**. Our data suggest that dysregulation of placental autophagy occurs more prominently during chronic immunopathology caused by *P*. *falciparum* in the context of placental malaria.

## Discussion

Each year, *P*. *falciparum* infection during pregnancy represents a burden that likely results in placental infection and consequent immunopathology that impacts placental homeostasis and fetal development [15]. Herein, we have shown that chronic and long-lasting immune responses and histopathology arising due to *P*. *falciparum* infection affect placental autophagy, which ultimately reflects impaired local homeostasis with consequences to gestational development.

The most frequent consequence of malaria in pregnancy (MiP) is the reduced birth weight due to preterm delivery and fetal growth restriction [25], and our results were in line with this. Although the etiology of this outcome is still unclear, evidence points to a plethora of insults that can range from exacerbated inflammation to deficient placental angiogenesis and reduced transplacental nutrient transport [15].

In our study, placentas derived from infected women presented a significant increase of syncytial nuclear aggregates and leukocytes infiltrate. These are formed predominantly by monocytes, known to be highly associated with reduced birth weight in newborns of *P*. *falciparum*-infected pregnant in malaria-endemic areas [9–12]. Regarding the cytokines profile, all placental pro-inflammatory cytokines presented similarities among both groups; though, IL-10, an anti-inflammatory cytokine, showed a striking augment in placentas from Pf-INF women. The IL-10 production during MiP-associated chronic inflammation is a hallmark of *falciparum* malaria during pregnancy that had been associated with poor pregnancy outcomes and considered as a possible MiP biomarker [26,27]. These chronic insults may persist despite inexistent parasitemia, which probably is associated with dysregulation of local homeostasis.

In MiP, placental monocytes infiltrate been shown to be predictive of autophagy dysfunction [19]. Autophagy is a homeostatic mechanism by which cells respond to stress induced by nutrient and oxygen scarcity, danger signals, exacerbated inflammation, and pathogens in order to subsist and survive [16,17]. During gestation, functional autophagy is pivotal to ensure proper fetal development in response to high nutritional demands and significant protein/cell turnover inside the placenta. Dysregulation of placental mammalian target of rapamycin (mTOR) signaling during placental malaria was suggested to be involved in the pathogenesis of reduced birth weight [28], through impaired transplacental nutrients transport [29,30]. In fact, alterations of this highly regulated homeostatic process are often associated with pregnancy complications such as preeclampsia, fetal growth restriction, and abortion [18]. As such, it was plausible that, in our samples, autophagy dysregulation could be associated with poor pregnancy outcomes. Interestingly, the evaluation of the ULK1, BECLIN1, and LC3 autophagy-related molecules revealed reduction of gene transcripts and decay of corresponding protein levels in placentas derived from *P*. *falciparum*-infected women.

Recently, findings from Dimasuay *et al*. revealed impaired placental autophagy in *P*. *falciparum*-infected Malawian women in which placental parasitemia and intervillositis (severe monocyte infiltrate) were present [19]. Their findings support that the impairment of autophagy arises due to the failure of autophagolysosome formation and cargo degradation despite an initial autophagic flux [19]. This study contrasts with ours, as in sub-Saharan Africa, where *P*. *falciparum*-infected women present placental parasitemia and severe intervillositis. Also, they show increased placental production of type 1 pro-inflammatory cytokines, such as TNF-α, IFN-γ, IL-1β, and IL-8, often accompanied by the unaltered or reduced production of type 2 cytokines, such as IL-10 [9–12]. Accordingly, placental autophagy is triggered to ensure homeostasis in the presence of acute inflammation, though, being functionally damaged due to deficient autophagosome-lysosome fusion [19].

On the other hand, in Brazil, few women present active/acute placental infection, as shown in our study. Our results showed that in the *P*. *falciparum*-infected women group, 15/43 were placental malaria negative (PM-) or 20/43 presented a past chronic placental malaria (PM+ by hemozoin detection but no detected parasitemia) [4,22]. Moreover, we have observed unaltered placental levels of type 1 cytokines and a significant increase in IL-10 levels, associated with the presence of leukocytes and frequent absence of parasitemia. Consequently, we showed that autophagy-related gene downregulation is more prominent in the Pf-PM+ group, which has higher levels of IL-10. The levels of TNF-α, IL-1β, IL-8, and IL-10 were negatively associated with *ULK1* transcription in placentas from Pf-INF women. Hence, our results suggest that autophagy is dysfunctional during placental malaria despite clear conflict between the regulatory nature of this process, which might be dependent on disease acuteness or chronicity.

In the last years, the role of distinct cytokines in the autophagy modulation has been addressed, suggesting that innate immunity signaling and type 1 cytokines induce autophagy while type 2 cytokines promote autophagy inhibition, particularly in the context of infection [17,31]. Therefore, it is possible to hypothesize that different cytokine patterns in distinct malaria endemicity settings may predict different autophagy profiles. Thus, reduced type 1 cytokines and significant production of IL-10 during MiP might downregulate placental autophagy.

Moreover, studies in pregnancy complications reported increased expression of autophagy-related genes, such as LC3B, in preeclampsia and fetal growth arrest [32–34]. On the other hand, studies in inflammation-derived preterm birth, of humans and mice, showed a reduction of autophagy molecules like LC3II, which is consensual with our results [35,36]. Nevertheless, that placental autophagy dysregulation arises, independently of the pregnancy insult etiology, changing the natural course of autophagy during gestation [33,37].

Remarkably, in our data, the autophagy dysregulation was more prominent when placental malaria was present. In fact, hemozoin, heme-derived pigment resulting from *P*. *falciparum* metabolism, was found to be negatively correlated with the autophagy-related genes transcript levels. *Per se*, the accumulation of hemozoin in the absence of infected erythrocytes may perpetuate the inflammation through the stimulus of immune cells and trophoblasts within the placenta [4,38,39]. However, the reduced number of placentas selected to the analyses of western blotting did not allow us to draw confident conclusions at the established level of significance.

Notwithstanding, our findings provide evidence on homeostasis imbalance during chronic placental malaria supported by autophagy dysregulation, raising concerns about disease treatment and its real impact on the resolution of placental malaria pathology.

## Supporting information

S1 Table Peripheral (1st infection and at delivery) and placental parasitemia from pregnant women infected with P. falciparum. Parasitemia of P. falciparum-infected women (43) who enrolled in the study measured by PET-PCR in the peripheral blood, first infection and at delivery, and in placental blood. Results are depicted as number of DNA copies. ID, participants identification in the study; Pf-INF PM-, placental malaria negative; Pf-INF PM+, placental malaria positive.
(DOCX)

S2 Table Spearman’s correlation analysis between placental autophagic-related genes mRNA levels and pregnancy histologic and immunologic parameters. Spearman correlation coefficients (r_s_) and *P* values generated from Spearman’s rank-order non-parametric test. BW–birth weight, PW–placental weight, HZ–hemozoin, SNA–syncytial nuclear aggregates, NECR–necrosis, VASC–vascularity, LEU–leukocytes, MO–monocytes.
(DOCX)

S1 Fig Levels of autophagy-related proteins are diminished in *P*. *falciparum*-infected placentas. Full-length and untreated images of western blots are shown for ULK1 (120 kDa) **(A)**, BECLIN1 (52 kDa) **(B)**, LC3I, and LC3II (16 and 14 kDa, respectively) **(C)**. A representative blotting for β-ACTIN (42 kDa) **(D)** is depicted, knowing that this endogenous control was performed in the same membrane as the corresponding protein of interest. Placental protein samples from non-infected (14) and *P*. *falciparum*-infected (14) women were randomly selected and separated in two different western blots gel/membrane and electrophoresis, blotting, and acquisition were performed simultaneously. Acquisition was performed at the ChemiDoc XRS+ at an exposure of 120.0 (ULK1), 30.0 (BECLIN1), 50.0 (LC3), and 20.0 (β-ACTIN) seconds. Molecular mass ladder is depicted for each gel (Precision Plus Protein^TM^ Standards, BIO-RAD).
(TIF)

S1 Dataset All values and measured presented. Please see excel file for documentation.
(XLSX)

S1 Raw Images Western blots original images. Full-length and untreated images of western blots are shown for ULK1 (120 kDa) **(A)**, BECLIN1 (52 kDa) **(B)**, LC3I, and LC3II (16 and 14 kDa, respectively) **(C)**. A representative blotting for β-ACTIN (42 kDa) **(D)** is depicted, knowing that this endogenous control was performed in the same membrane as the corresponding protein of interest. Placental protein samples from non-infected (14) and *P*. *falciparum*-infected (14) women were randomly selected and separated in two different western blots gel/membrane and electrophoresis, blotting, and acquisition were performed simultaneously. Acquisition was performed at the ChemiDoc XRS+ at an exposure of 120.0 (ULK1), 30.0 (BECLIN1), 50.0 (LC3), and 20.0 (β-ACTIN) seconds. Molecular mass ladder is depicted for each gel (Precision Plus Protein^TM^ Standards, BIO-RAD).
(PDF)
