# Supplementary material for: Plasmodium falciparum infection dysregulates placental autophagy
Source: PLoS One. 2019 Dec 5;14(12):e0226117. doi: 10.1371/journal.pone.0226117 (PMC6894763; doi:10.1371/journal.pone.0226117)
Supplement: S1 Table — Parasitemia of P. falciparum-infected women (43) who enrolled in the study measured by PET-PCR in the peripheral blood, first infection and at delivery, and in placental blood. Results are depicted as number of DNA copies. ID, participants identification in the study; Pf-INF PM-, placental malaria negative; Pf-INF PM+, placental malaria positive. (DOCX) [file pone.0226117.s001.docx]

**S1 Table. Peripheral (1^st^ infection and at delivery) and placental parasitemia from pregnant women infected with *P. falciparum.***

| **ID** | **PM Status** | **Parasitemia 1º Infection** | **Parasitemia at Delivery** | **Placental Parasitemia** |
| --- | --- | --- | --- | --- |
| **3** | Pf-INF PM- | 23759.20 | . | . |
| **7** | Pf-INF PM- | 37733.70 | . | . |
| **46** | Pf-INF PM- | 19.50 | 6.70 | 1.90 |
| **58** | Pf-INF PM- | 1.90 | . | . |
| **77** | Pf-INF PM- | 651.60 | . | . |
| **140** | Pf-INF PM- | 58.50 | . | . |
| **241** | Pf-INF PM- | 903.60 | . | . |
| **287** | Pf-INF PM- | 0.50 | . | . |
| **338** | Pf-INF PM- | 681.20 | . | . |
| **344** | Pf-INF PM- | 11990.00 | . | . |
| **350** | Pf-INF PM- | 385.40 | . | . |
| **417** | Pf-INF PM- | 1499.00 | . | . |
| **451** | Pf-INF PM- | 1408.00 | . | . |
| **504** | Pf-INF PM- | 4161.00 | . | 0.50 |
| **528** | Pf-INF PM- | 8754.00 | . | . |
| **4** | Pf-INF PM+ | 672.70 | 696.70 | 63.80 |
| **70** | Pf-INF PM+ | 1372.00 | 1372.00 | 1818.70 |
| **248** | Pf-INF PM+ | 561.40 | 561.40 | 417.50 |
| **416** | Pf-INF PM+ | 4758.00 | 4758.00 | 22580.00 |
| **461** | Pf-INF PM+ | 9046.00 | . | 62.10 |
| **582** | Pf-INF PM+ | 61120.00 | 61120.00 | 63440.00 |
| **130** | Pf-INF PM+ | 5195.00 | 5195.00 | 21460.00 |
| **394** | Pf-INF PM+ | 1.50 | . | 900.00 |
| **20** | Pf-INF PM+ | 2989.20 | . | . |
| **21** | Pf-INF PM+ | 1957.80 | . | . |
| **90** | Pf-INF PM+ | 1362.00 | . | . |
| **94** | Pf-INF PM+ | 3237.00 | . | . |
| **105** | Pf-INF PM+ | 15840.00 | . | . |
| **124** | Pf-INF PM+ | 1754.00 | . | . |
| **262** | Pf-INF PM+ | 242.10 | . | . |
| **278** | Pf-INF PM+ | 414.70 | . | . |
| **289** | Pf-INF PM+ | 4503.00 | . | . |
| **326** | Pf-INF PM+ | 15970.00 | . | . |
| **335** | Pf-INF PM+ | 86.50 | . | . |
| **339** | Pf-INF PM+ | 3031.00 | 1.90 | 0.90 |
| **358** | Pf-INF PM+ | 2682.00 | . | . |
| **402** | Pf-INF PM+ | 2971.00 | . | . |
| **422** | Pf-INF PM+ | 3051.00 | . | . |
| **424** | Pf-INF PM+ | 277.70 | . | . |
| **433** | Pf-INF PM+ | 4553.00 | . | . |
| **488** | Pf-INF PM+ | 502.00 | . | . |
| **507** | Pf-INF PM+ | 15430 | . | . |
| **512** | Pf-INF PM+ | 5111.00 | . | . |

Parasitemia of *P. falciparum*-infected women (43) who enrolled in the study measured by PET-PCR in the peripheral blood, first infection and at delivery, and in placental blood. Results are depicted as number of DNA copies. ID, participants identification in the study; Pf-INF PM-, placental malaria negative; Pf-INF PM+, placental malaria positive.
