# Supplementary material for: Plasmodium falciparum infection dysregulates placental autophagy
Source: PLoS One. 2019 Dec 5;14(12):e0226117. doi: 10.1371/journal.pone.0226117 (PMC6894763; doi:10.1371/journal.pone.0226117)
Supplement: S2 Table — Spearman correlation coefficients (rs) and P values generated from Spearman’s rank-order non-parametric test. BW–birth weight, PW–placental weight, HZ–hemozoin, SNA–syncytial nuclear aggregates, NECR–necrosis, VASC–vascularity, LEU–leukocytes, MO–monocytes. (DOCX) [file pone.0226117.s002.docx]

**S2 Table. Spearman’s correlation analysis between placental autophagic-related genes mRNA levels and pregnancy histologic and immunologic parameters.**

|  | **NON-INFECTED** | | | | | | ***P. falciparum*-INFECTED** | | | | | |
| --- | --- | --- | --- | --- | --- | --- | --- | --- | --- | --- | --- | --- |
|  | ***ULK1*** | | ***BECN1*** | | ***MAP1LC3B*** | | ***ULK1*** | | ***BECN1*** | | ***MAP1LC3B*** | |
|  | **r_s_** | ***P* value** | **r_s_** | ***P* value** | **r_s_** | ***P* value** | **r_s_** | ***P* value** | **r_s_** | ***P* value** | **r_s_** | ***P* value** |
| **BW** | -0.05 | 0.7454 | -0.05 | 0.7369 | 0.05 | 0.7644 | -0.01 | 0.9553 | 0.02 | 0.9132 | 0.07 | 0.6503 |
| **PW** | -0.11 | 0.4789 | -0.02 | 0.8987 | -0.07 | 0.6669 | 0.15 | 0.3568 | -0.24 | 0.1404 | 0.00 | 0.9961 |
| **HZ** | . | . | . | . | . | . | ***-0.57*** | ***< 0.0001*** | ***-0.40*** | ***0.0088*** | ***-0.42*** | ***0.0053*** |
| **SNA** | 0.05 | 0.7357 | -0.05 | 0.7673 | 0.02 | 0.8874 | -0.23 | 0.1494 | -0.28 | 0.0718 | ***-0.32*** | ***0.0378*** |
| **FIBRIN** | -0.11 | 0.4914 | 0.06 | 0.7004 | -0.01 | 0.9632 | -0.23 | 0.1442 | -0.20 | 0.2008 | -0.27 | 0.0822 |
| **NECR** | 0.10 | 0.545 | 0.03 | 0.8649 | 0.14 | 0.386 | 0.10 | 0.5162 | 0.12 | 0.4391 | 0.22 | 0.1556 |
| **VASC** | 0.14 | 0.4025 | 0.10 | 0.5471 | 0.07 | 0.6548 | -0.15 | 0.3762 | -0.26 | 0.1252 | -0.28 | 0.0904 |
| **LEU** | 0.05 | 0.7407 | -0.22 | 0.1788 | -0.03 | 0.8494 | -0.17 | 0.2961 | -0.23 | 0.1428 | -0.24 | 0.1267 |
| **MO** | 0.20 | 0.2082 | 0.15 | 0.3534 | 0.21 | 0.1878 | 0.04 | 0.8203 | -0.01 | 0.9307 | 0.19 | 0.2239 |
| **IL-12** | -0.27 | 0.0848 | -0.12 | 0.4498 | -0.13 | 0.3942 | -0.24 | 0.1456 | -0.09 | 0.6069 | -0.08 | 0.6243 |
| **IL-8** | 0.03 | 0.8411 | 0.17 | 0.3222 | 0.03 | 0.8756 | ***-0.37*** | ***0.0276*** | -0.02 | 0.9135 | -0.05 | 0.7667 |
| **TNF-α** | ***-0.34*** | ***0.0311*** | -0.10 | 0.5514 | -0.27 | 0.086 | ***-0.37*** | ***0.0300*** | -0.02 | 0.9119 | -0.10 | 0.5685 |
| **IL-10** | -0.20 | 0.2265 | -0.12 | 0.4679 | -0.07 | 0.6871 | ***-0.36*** | ***0.0356*** | 0.03 | 0.8724 | -0.12 | 0.4726 |
| **IL-6** | -0.06 | 0.7387 | 0.13 | 0.4667 | 0.01 | 0.9492 | ***-0.39*** | ***0.0199*** | 0.05 | 0.7804 | -0.06 | 0.7175 |
| **IL-1β** | -0.07 | 0.6829 | 0.24 | 0.1464 | 0.02 | 0.89 | -0.30 | 0.084 | -0.05 | 0.7674 | -0.10 | 0.5783 |

Spearman correlation coefficients (r_s_) and *P* values generated from Spearman’s rank-order non-parametric test. BW – birth weight. PW – placental weight. HZ – hemozoin. SNA – syncytial nuclear aggregates. NECR – necrosis. VASC – vascularity. LEU – leukocytes. MO – monocytes.
