# Supplementary figures and images for: Plasmodium falciparum infection dysregulates placental autophagy
Source: PLoS One. 2019 Dec 5;14(12):e0226117. doi: 10.1371/journal.pone.0226117 (PMC6894763; doi:10.1371/journal.pone.0226117)

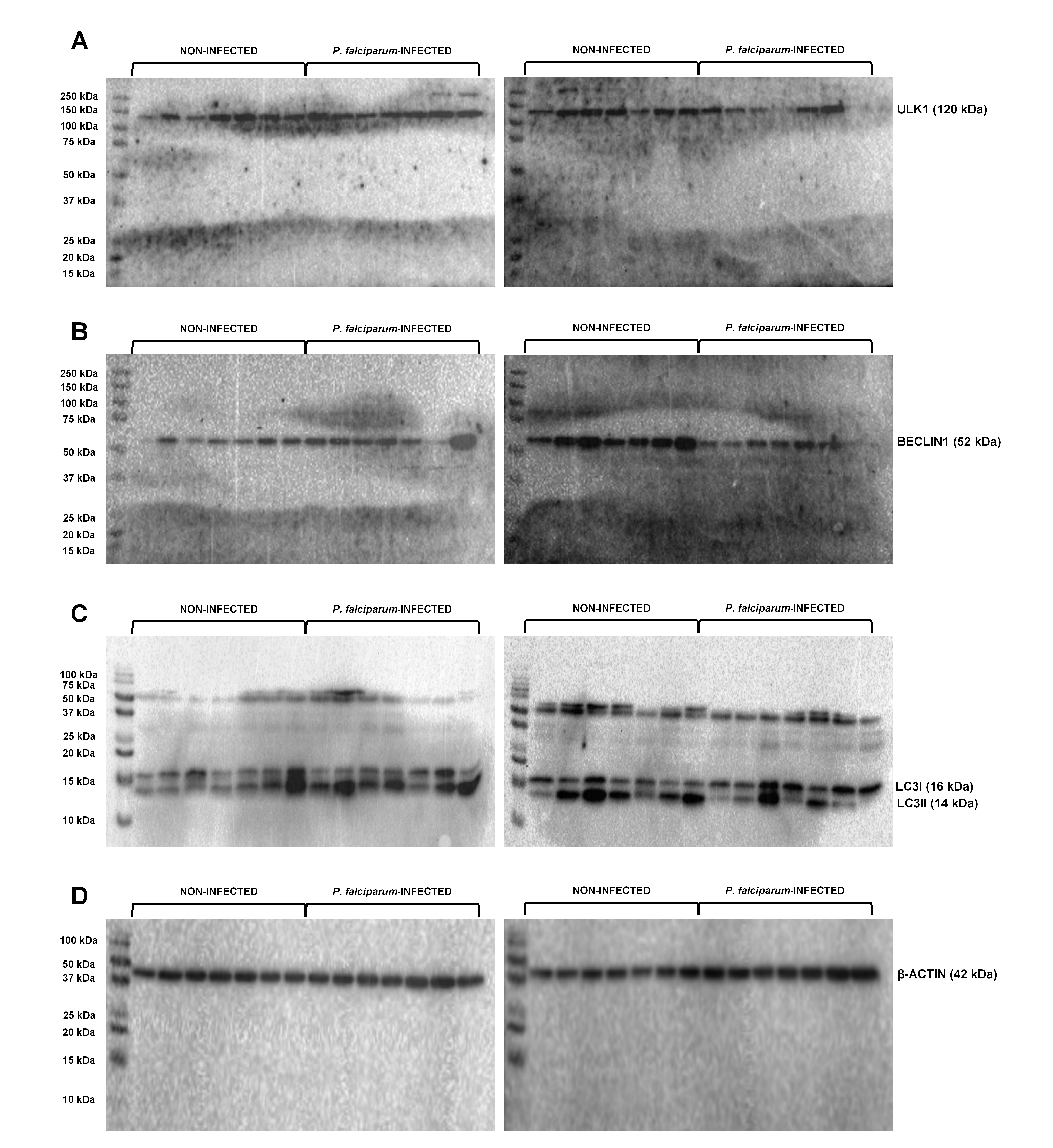

Supplement: S1 Fig — Full-length and untreated images of western blots are shown for ULK1 (120 kDa) (A), BECLIN1 (52 kDa) (B), LC3I, and LC3II (16 and 14 kDa, respectively) (C). A representative blotting for β-ACTIN (42 kDa) (D) is depicted, knowing that this endogenous control was performed in the same membrane as the corresponding protein of interest. Placental protein samples from non-infected (14) and P. falciparum-infected (14) women were randomly selected and separated in two different western blots gel/membrane and electrophoresis, blotting, and acquisition were performed simultaneously. Acquisition was performed at the ChemiDoc XRS+ at an exposure of 120.0 (ULK1), 30.0 (BECLIN1), 50.0 (LC3), and 20.0 (β-ACTIN) seconds. Molecular mass ladder is depicted for each gel (Precision Plus ProteinTM Standards, BIO-RAD). (TIF) [file pone.0226117.s003.tif]
