# Supplementary material for: Plasmodium falciparum infection dysregulates placental autophagy
Source: PLoS One. 2019 Dec 5;14(12):e0226117. doi: 10.1371/journal.pone.0226117 (PMC6894763; doi:10.1371/journal.pone.0226117)
Supplement: S1 Raw Images — Full-length and untreated images of western blots are shown for ULK1 (120 kDa) (A), BECLIN1 (52 kDa) (B), LC3I, and LC3II (16 and 14 kDa, respectively) (C). A representative blotting for β-ACTIN (42 kDa) (D) is depicted, knowing that this endogenous control was performed in the same membrane as the corresponding protein of interest. Placental protein samples from non-infected (14) and P. falciparum-infected (14) women were randomly selected and separated in two different western blots gel/membrane and electrophoresis, blotting, and acquisition were performed simultaneously. Acquisition was performed at the ChemiDoc XRS+ at an exposure of 120.0 (ULK1), 30.0 (BECLIN1), 50.0 (LC3), and 20.0 (β-ACTIN) seconds. Molecular mass ladder is depicted for each gel (Precision Plus ProteinTM Standards, BIO-RAD). (PDF) [file pone.0226117.s005.pdf]

Plasmodium falciparum infection dysregulates placental autophagy

Flávia A. Lima[1,¶], André Barateiro[1,¶], Jamille G. Dombrowski[1], Rodrigo M. de Souza[2], Douglas S. Costa[1], Oscar Murillo[1], Sabrina Epiphanio[3], Lígia A. Gonçalves[1,&,*], Claudio R. F. Marinho[1,&,*]

[1]Department of Parasitology, Institute of Biomedical Sciences, University of São Paulo, São Paulo, Brazil

[2]Multidisciplinary Centre, Federal University of Acre, Acre, Brazil

[3]Department of Clinical and Toxicological Analysis, School of Pharmaceutical Sciences, University of São Paulo, São Paulo, Brazil

*Corresponding authors:

E-mail: marinho@usp.br (CRFM) and lig.antunes.goncalves@gmail.com

[¶]These authors contributed equally to this work

[&]These authors also contributed equally to this work

Short title: *P. falciparum* affects placental autophagy.

S1 Raw Images. Full-length and untreated images of western blots are shown for ULK1 (120 kDa), BECLIN1 (52 kDa), LC3I, and LC3II (16 and 14 kDa, respectively) and a representative blotting for β-ACTIN (42 kDa) is depicted knowing that this endogenous control was performed in the same membrane as the corresponding protein of interest. Placental protein samples from non-infected (14) and *P. falciparum*-infected (14) women were randomly selected and separated in two different western blots gel/membrane and electrophoresis, blotting, and acquisition were performed simultaneously. Herein, we show the samples/lanes identified with a number from 1 to 28, and a “X” identifying the lanes that were not used in the Fig 2A of the manuscript. Acquisition was performed for both gels of the same marker at the same time using the ChemiDoc XRS+ at an exposure of 120.0 (ULK1), 30.0 (BECLIN1), 50.0 (LC3) and 20.0 (β-ACTIN) seconds. Molecular mass ladder is depicted for each gel (Precision Plus Protein^{TM} Standards, BIO-RAD).

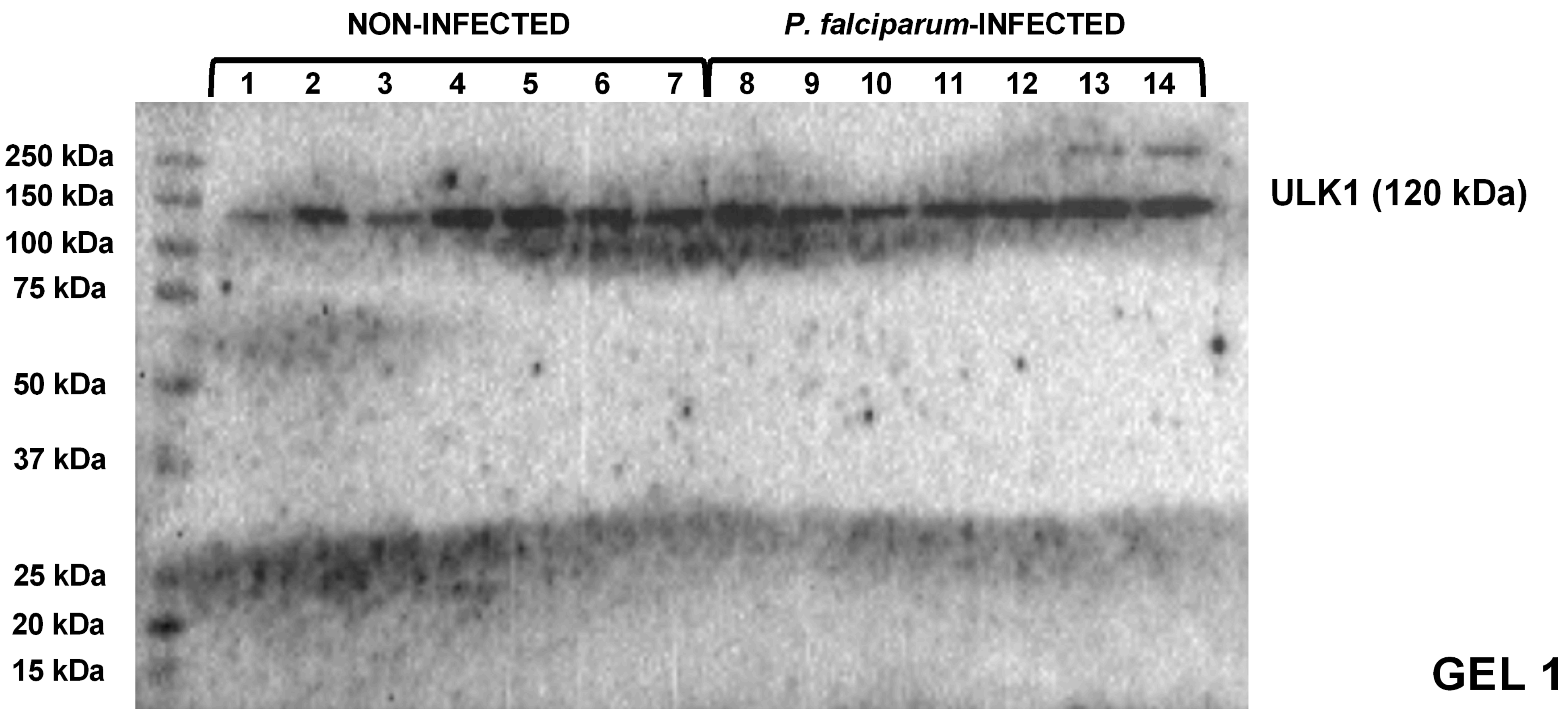
NON-INFECTED
P. falciparum-INFECTED
1
2
3
4
5
6
7
8
9
10
11
12
13
14
250 kDa
150 kDa
100 kDa
75 kDa
50 kDa
37 kDa
25 kDa
20 kDa
15 kDa
ULK1 (120 kDa)
GEL 1

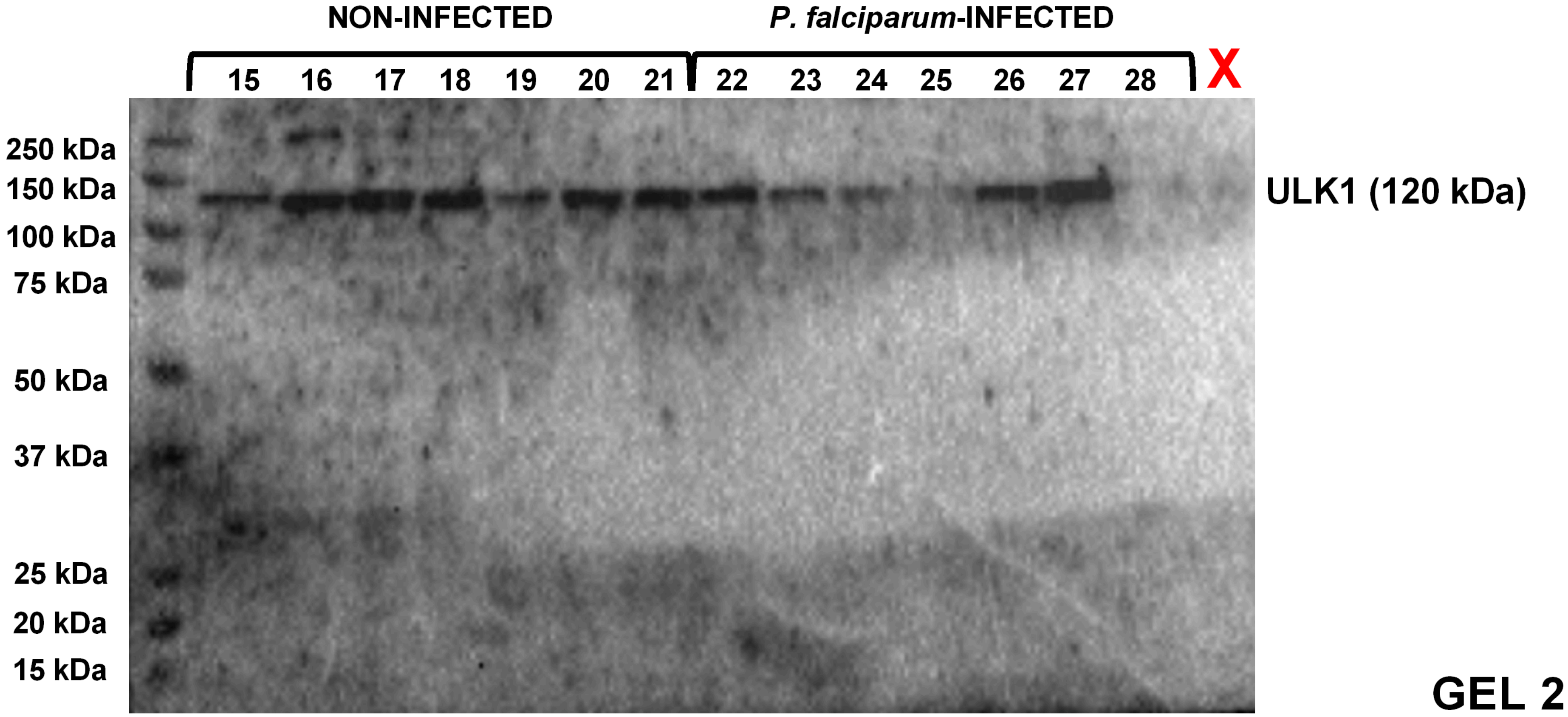
NON-INFECTED
P. falciparum-INFECTED
15
16
17
18
19
20
21
22
23
24
25
26
27
28
X
250 kDa
150 kDa
100 kDa
75 kDa
50 kDa
37 kDa
25 kDa
20 kDa
15 kDa
ULK1 (120 kDa)
GEL 2

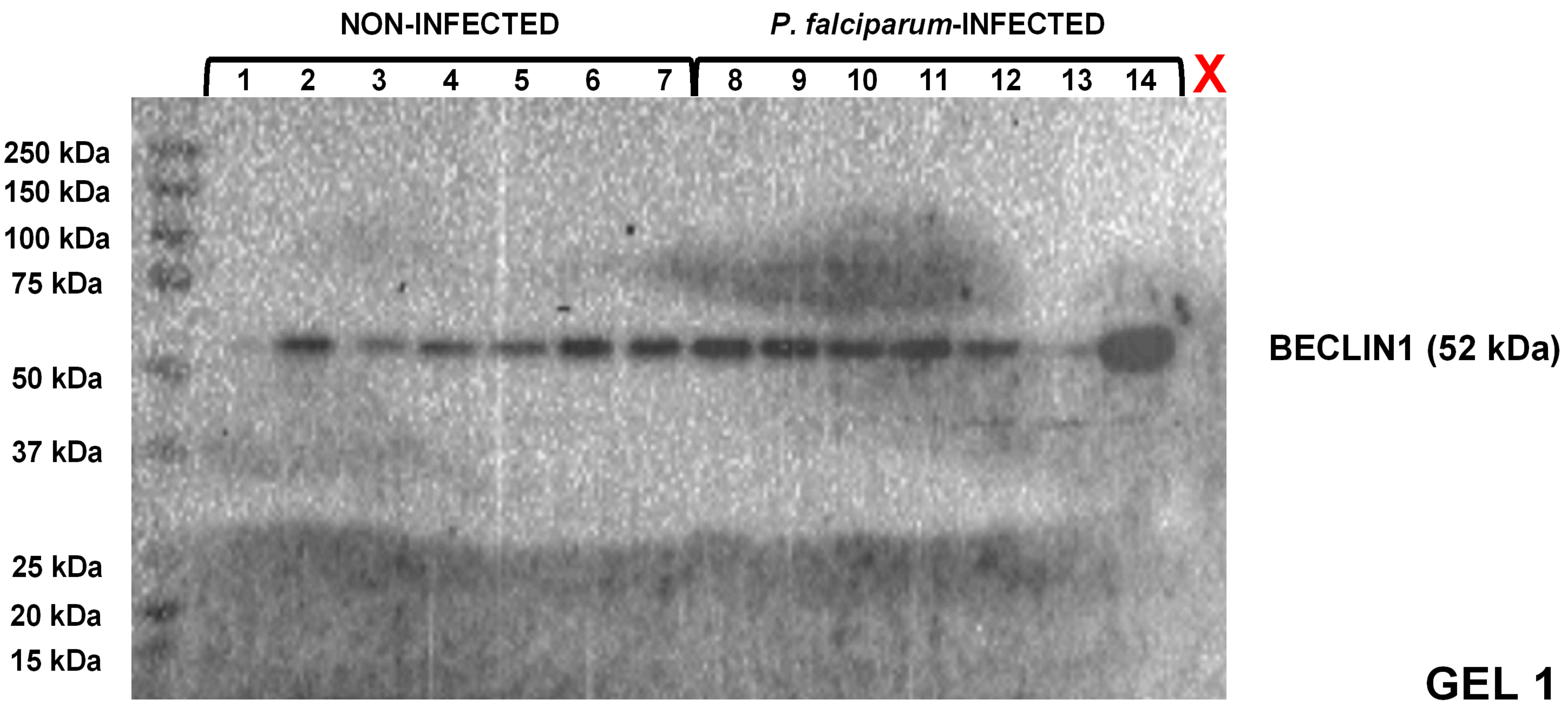
NON-INFECTED
P. falciparum-INFECTED
1
2
3
4
5
6
7
8
9
10
11
12
13
14
X
250 kDa
150 kDa
100 kDa
75 kDa
50 kDa
37 kDa
25 kDa
20 kDa
15 kDa
BECLIN1 (52 kDa)
GEL 1

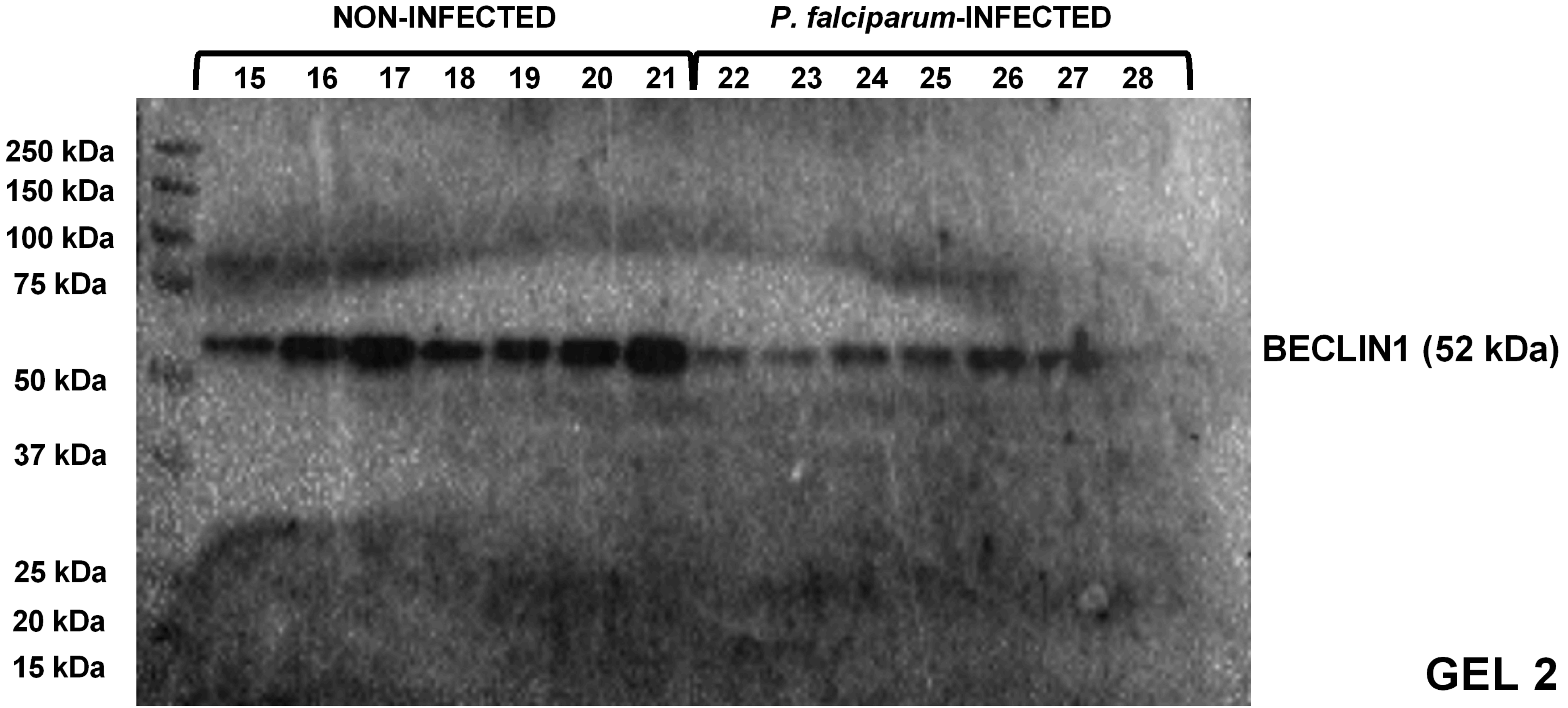
NON-INFECTED
P. falciparum-INFECTED
15
16
17
18
19
20
21
22
23
24
25
26
27
28
250 kDa
150 kDa
100 kDa
75 kDa
50 kDa
37 kDa
25 kDa
20 kDa
15 kDa
BECLIN1 (52 kDa)

GEL 2

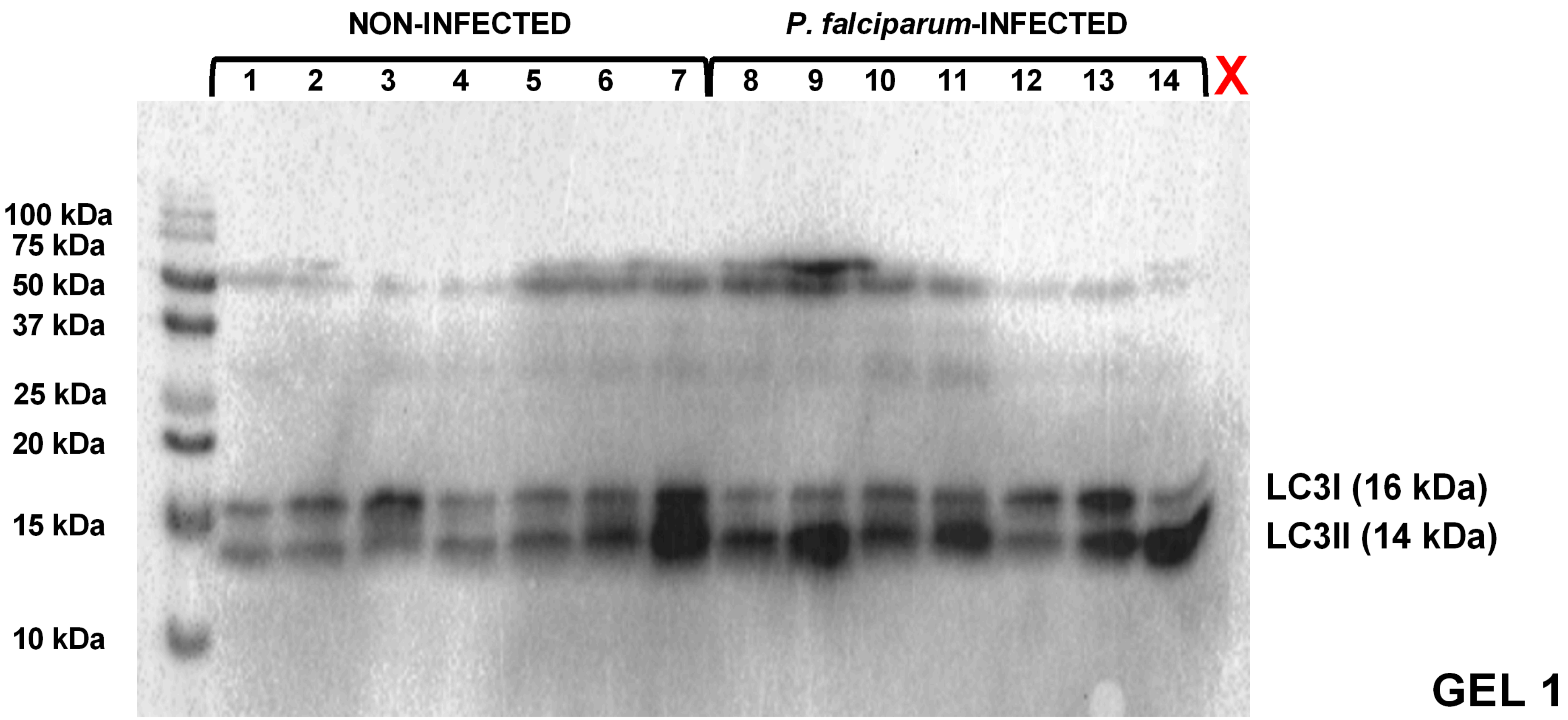
NON-INFECTED
P. falciparum-INFECTED
1
2
3
4
5
6
7
8
9
10
11
12
13
14
X
100 kDa
75 kDa
50 kDa
37 kDa
25 kDa
20 kDa
15 kDa
10 kDa
LC3I (16 kDa)
LC3II (14 kDa)
GEL 1

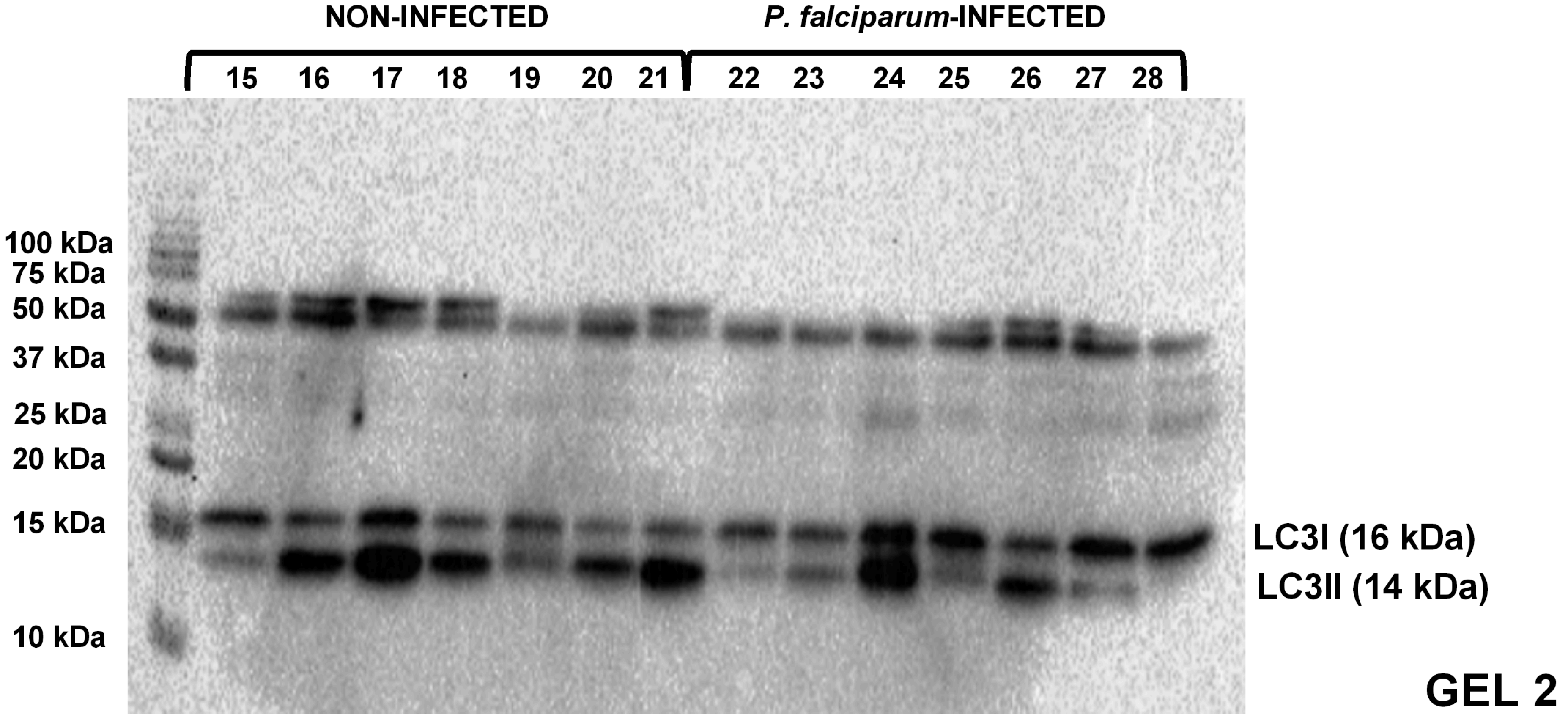
NON-INFECTED
P. falciparum-INFECTED
15 16 17 18 19 20 21 22 23 24 25 26 27 28
100 kDa
75 kDa
50 kDa
37 kDa
25 kDa
20 kDa
15 kDa
10 kDa
LC3I (16 kDa)
LC3II (14 kDa)
GEL 2

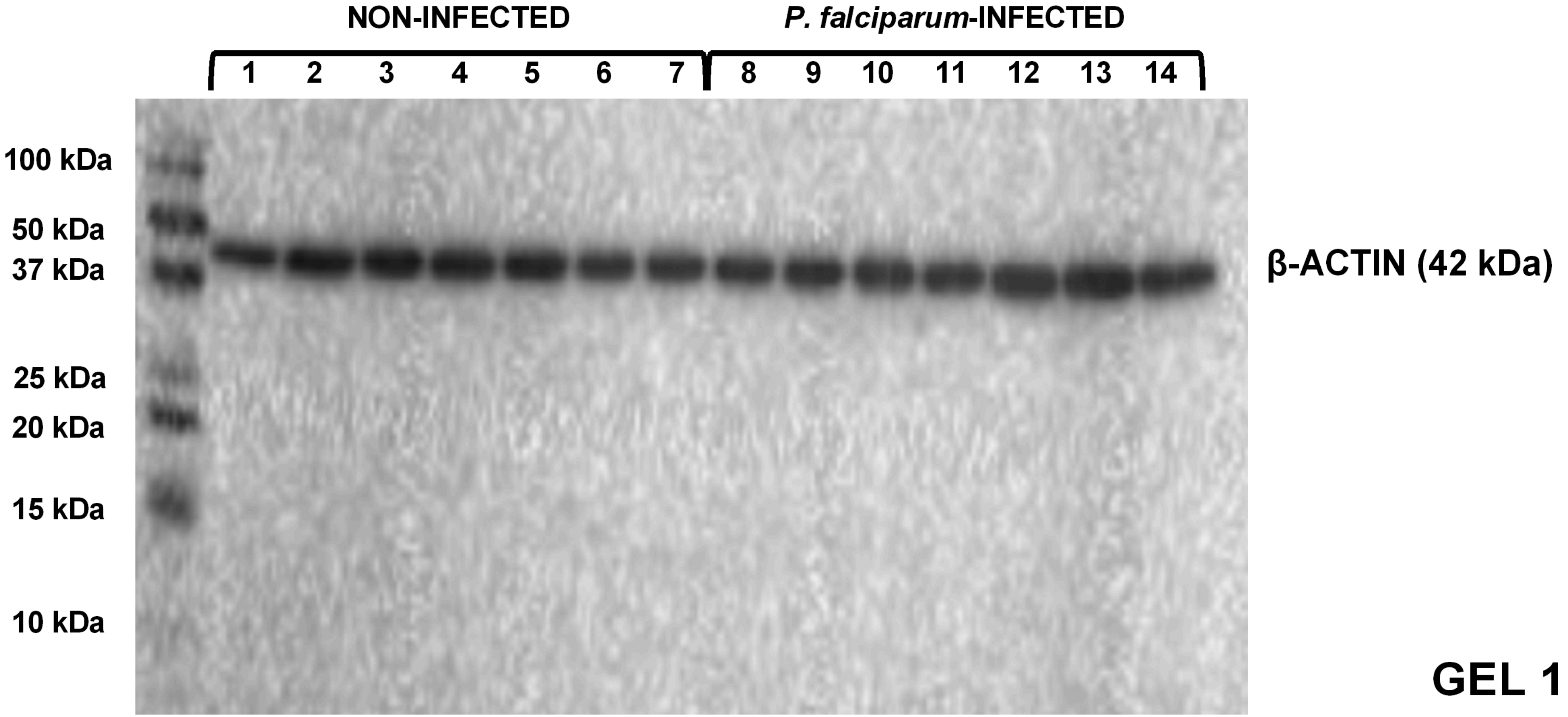
NON-INFECTED
P. falciparum-INFECTED
1
2
3
4
5
6
7
8
9
10
11
12
13
14
100 kDa
50 kDa
37 kDa
25 kDa
20 kDa
15 kDa
10 kDa
β-ACTIN (42 kDa)
GEL 1

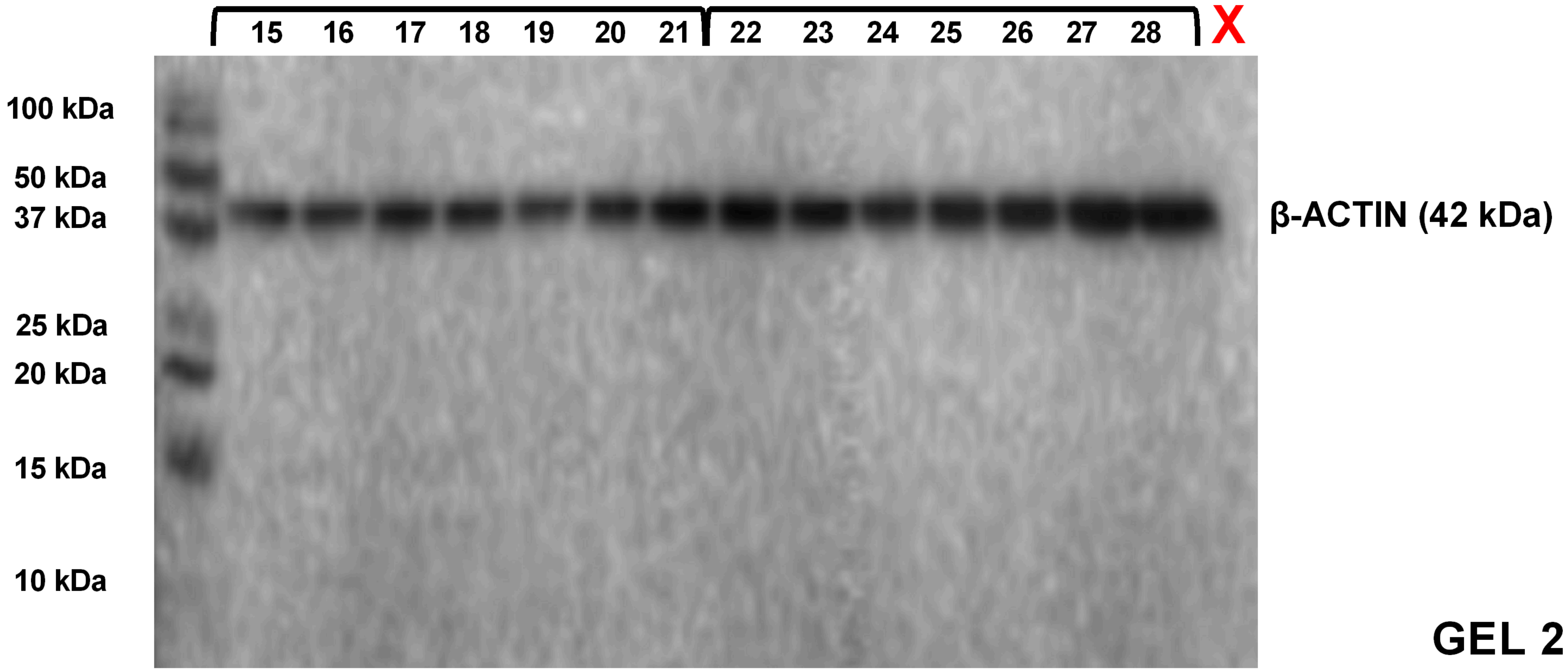

15
16
17
18
19
20
21
22
23
24
25
26
27
28
X
100 kDa
50 kDa
37 kDa
25 kDa
20 kDa
15 kDa
10 kDa
β-ACTIN (42 kDa)
GEL 2
